# *Fusobacterium nucleatum* Extracellular Vesicles Modulate Gut Epithelial Cell Innate Immunity *via* FomA and TLR2

**DOI:** 10.3389/fimmu.2020.583644

**Published:** 2020-12-21

**Authors:** Camille Martin-Gallausiaux, Antoine Malabirade, Janine Habier, Paul Wilmes

**Affiliations:** ^1^ Luxembourg Centre for Systems Biomedicine, University of Luxembourg, Esch-sur-Alzette, Luxembourg; ^2^ Department of Life Sciences and Medicine, Faculty of Science, Technology and Medicine, University of Luxembourg, Esch-sur-Alzette, Luxembourg

**Keywords:** Fusobacterium, extracellular vesicle, innate immunity, gut microbiota, intestinal epithelial cell, Toll-like receptor 2

## Abstract

Extracellular vesicles (EVs) derived from the gut microbiota are largely uncharacterized and their impacts on host intestinal physiology remain unresolved. Here, we isolated EVs from *F. nucleatum* for detailed characterization. Our analyses highlight the presence of the outer membrane protein porin FomA on EVs. Besides, we evaluated the impact of EVs on human intestinal epithelial cells (IECs) in a non-inflammatory context. Our results show no detrimental impact on the epithelial barrier. No internalization of EVs was observed. Moreover, we demonstrate that *F. nucleatum* EVs trigger innate immunity of IECs by promoting NF-κB activation *via* the dynamin-mediated endocytosis. The NF-κB activation was found to be TLR2-dependent yet, TLR4 was dispensable. Using competitive binding assays, we establish that FomA is involved in the NF-κB response. Taken together, our data indicate that EVs induce effects similar to those observed with whole *F. nucleatum* bacteria on IECs. In particular, our study highlights the role of TLR2 and FomA as major modulators of the gut epithelium immune responses to *F. nucleatum*.

**Graphical Abstract:**
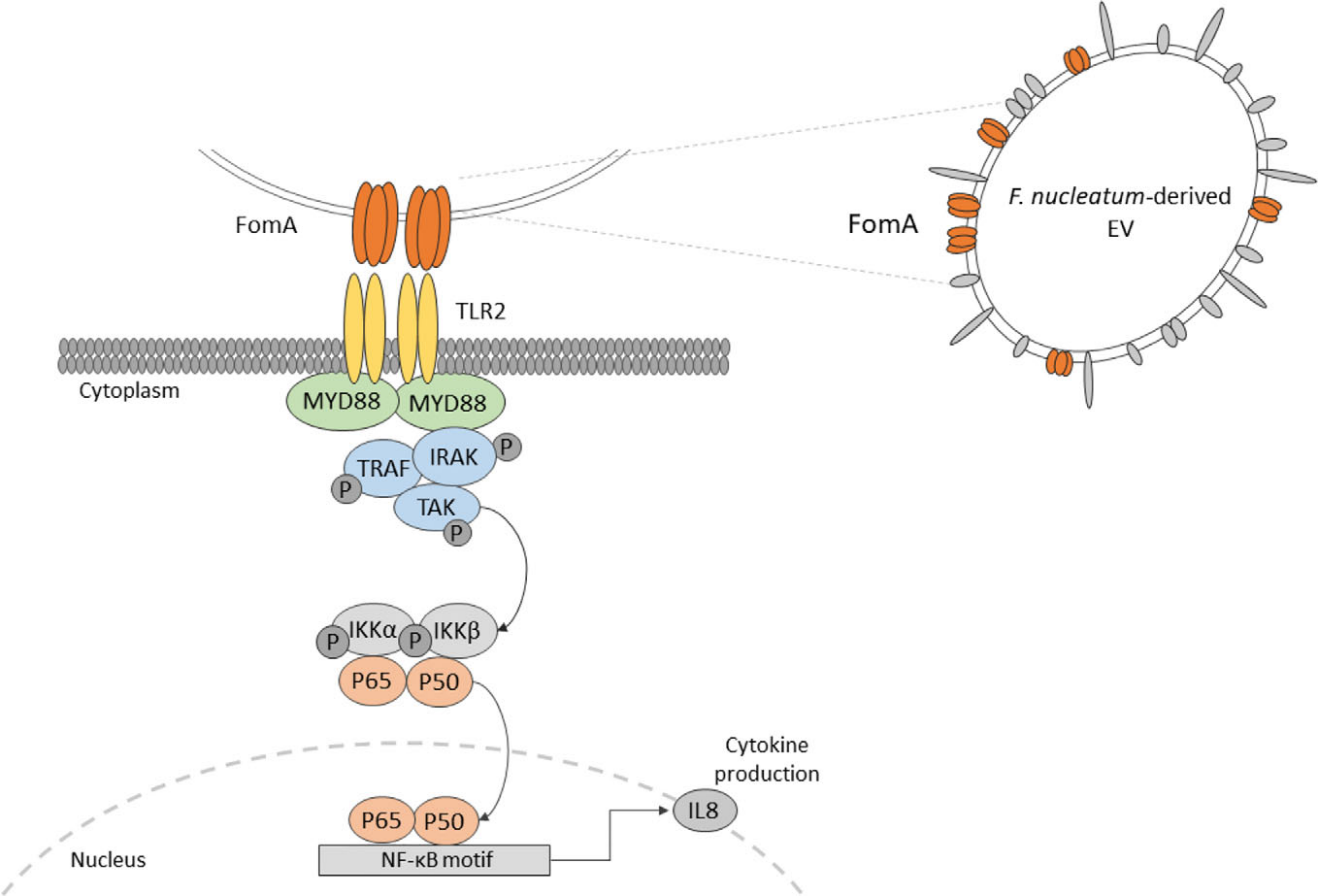


## Introduction

The gut microbiota and its human host are constantly exchanging a panoply of distinct molecules, resulting in a complex and dynamic interrelationship. This molecular dialogue is dependent on the microbial composition at the strain level and functions at gene level, and shifts in the microbial community can have profound effects on the host’s health status. However, alterations of community composition are context- and disease-specific, and rarely homogenous across patients leading to unsolved questions over causality (1, 2). Despite these heterogeneities, the over-growth of species expressing a high “pathogenic potential”, previously referred to as pathobionts, is one feature repeatedly associated with alterations of the gut microbiota composition (3). Several symbiotic species are able to disrupt host defences in a susceptible host, such as *Helicobacter hepaticus*, Segmented filamentous bacteria (SFB), *Escherichia coli* or *Fusobacterium nucleatum* (4, 5). *F. nucleatum* is an interesting case: in a “healthy” human gut microbiota, this bacterium is often present at low abundance and produces large amounts of beneficial short chain fatty acids (6). Yet, in periodontal diseases or colorectal cancers, the bacterium expands in a subset of individuals (7–10). *F. nucleatum* increased abundance in colonic biopsies is associated with a shorter survival and resistance to chemotherapy in colorectal cancer (11, 12). However, what triggers *Fusobacterium* expansion in these individuals is undetermined.

A common characteristic of pathobionts is the production of immunomodulatory molecules, which induce potent innate immune responses at the level of the intestinal mucosa. *F. nucleatum* produces several known virulence factors, involved in inflammation among which some are linked to oncogenesis. Adhesins such as FadA binds to E-cadherin expressed by epithelial and endothelial cells and activates the β-catenin/Wnt pathway (13, 14). The bacterium also expresses Fap2, a large protein involved in tumor cell adhesion which are expressing a high level of D-galactose-β (1–3)-N-acetyl-D-galactosamine (Gal-GalNAc) polysaccharide. Fap2 adhesion favors bacterial internalization in non-phagocytic cells (15, 16). Apart from Fap2, the non-specific porin FomA is one of the most expressed outer membrane proteins (OMP), representing around 30% of such proteins. FomA also has a role in cell adhesion and has immunogenic properties (17, 18). Overall, the molecular mechanisms by which *F. nucleatum* interacts with host colonocytes and modulate innate immunity are poorly characterized, particularly in homeostatic conditions. *F. nucleatum* activates Toll-like receptors (TLRs), particularly TLR2 and TLR4 in periodontal, immune and intestinal epithelial cells (IECs), and thereby to induce an NF-κB immune response (19–21).

In addition to cell-bacteria interactions, extracellular vesicles (EVs) produced by *F. nucleatum* may also contribute to host immune-regulation. EVs are thought to be universally released in all domains of life, including by bacteria. They are produced by blebbing of the bacterial membrane and can include all the internal components of a bacterial cell: nucleic acids, proteins, lipids, and sugars (22). Based on their contents, vesicles play crucial roles in host-microbe interactions as modulator of host cell functions with different molecules including virulence factors. Several studies already demonstrated the role of some bacterial EVs in the immune regulation of the host, however EVs derived from *F. nucleatum* have never been evaluated (23, 24). In this context, we aim to investigate *F. nucleatum*-derived EVs composition and impact in regulating IECs innate immune response at steady state.

Here, we isolated and characterized highly purified *F. nucleatum* subsp *nucleatum*-derived EVs. We quantified the concentration of EVs, their size, as well as study the EVs’ protein and LPS contents. Furthermore, we demonstrate the presence of the porin FomA on *F. nucleatum* EVs. *In vitro*, EVs have no detrimental impact on the epithelial intestinal barrier and we did not observed EVs internalization. Moreover, we investigated the impact of *F. nucleatum* EVs on the innate immune response and show that EVs promote a strong NF-κB response which was TLR2-dependent and rely on dynamin-mediated endocytosis. Furthermore, using a competitive binding inhibition test, we demonstrate that FomA is involved in the EVs-dependent NF-κB response. Our results show that EVs effects are similar to those induced by whole bacteria. Overall, our study advances our understanding of the molecular mechanism underlying *F. nucleatum* EVs interactions with the gut epithelium.

## Material and Methods

### Reagents

Ultrapure LPS (100 µg.ml^−1^ to 10 µg.ml^−1^) from *E. coli* 055:B5 (Invivogen, # tlrl-pb5lps), 1 µg.ml^−1^ Pam3CSK4 (Invivogen, # tlrl-pms), 10 ng.ml^−1^ IL−1β, and 10 ng.ml^−1^ TNF-α (Peprotech, #200-01B and 300-01A respectively). Inhibitors were added 2 h prior testing: 1 µM TAK-242 TLR4 inhibitor (Calbiochem, # 243984-11-4), 20 µM Dynasore (Sigma-Aldrich, # 324410), 1 µM CU-CTP22 TLR1/TLR2 antagonist (Merck Millipore # 614305), Genistein 50 µM (Abcam # ab120112), Wortmannin 20µM (Abcam # ab120148), Chlorpromazine hydrochloride (Sigma # C0982), PitStop2 (Sigma # SML 1169), and Casin (Sigma # SML 1253).

### Human Cell Lines

HCT116 (ATCC), HT-29 (ECACC) were maintained in RPMI 1640 (Gibco) supplemented with 10% FBS (Gibco), 1% Penicillin -Streptomycin, 1% non-essential amino acids (Gibco), 1 mM Sodium pyruvate (Gibco) and 10 mM HEPES (Sigma) in a 5% CO_2_ incubator. Thp1 XBlue, HEK Null1, TLR2, TLR4, NOD1, and NOD2 bearing NFkB-AP1 reporter (Invivogen) were maintained in the same conditions. Caco-2 (ATCC) were cultured in DMEM supplemented with 10% FBS (Difco), 1% Penicillin-Streptomycin (Gibco), 1% MEM non-essential amino acids (Gibco), 1 mM Sodium pyruvate (Gibco), and 10 mM HEPES (Sigma) in a 10% CO_2_. T84 (ATCC) were grown in DMEM-F12 medium supplemented with 10% FBS (Gibco), 1% Penicillin-Streptomycin (Gibco), 1% MEM non-essential amino acids (Gibco), 1 mM Sodium pyruvate (Gibco), and 10 mM HEPES (Sigma) in a 5% CO_2_ incubator. Mycoplasma and bacterial contaminations were tested regularly by PCR or using HEK TLR2 cell-line. Cell viability was monitored by MTS measurement using the CellTiter 96 Aqueous One solution (Promega) according to the manufacturer’s recommendations.

#### NF-κB Reporter Cells Lines

Caco-2, HCT116, and T84 cells were transfected with pNifty2-NF-κB SEAP plasmid (Invivogen) using Lipofectamine 3000 (Invitrogen) and selected with Zeocin (Invivogen) for at least 8 weeks and sub-cloned to establish a stable monoclonal cell-line. For each experiment, reporter cells were seeded at 30,000 cells per well in a 96 well plate 24 h prior stimulation in technical duplicate. After 24 h of incubation, 20 µl of supernatant was added to 180 µl of Quanti-Blue substrate (Invivogen) and read at 655 nM using Synergy Mx Microplate reader (Biotek Instruments). All experiments were reproduced at least three times using at least two different EVs extractions.

#### siRNA Assay

T84 NF-κB cells were seeded at 10,000 cells per well in a 96 wells plate. The next day siRNA transfection was prepared by mixing 0.5 µl.well 5 µM siRNA control (ON-TARGETplus Non-targeting Control Pool D-001810-10-05) in Optimem or 0.5 µl.well 5 µM siRNA TLR2 (ON-TARGETplus Human TLR2 (7097) siRNA - SMARTpool L-005120-01-0005) or 0.5µl.well 5µM siRNA TLR4 (ON-TARGETplus Human TLR4 (7099) siRNA - SMARTpool L-008088-01-0005) and 0.2 µl.well DharmaFECT reagent 1. After 5 min of incubation siRNA and transfection reagent were mixed and left for 20-min incubation at room temperature before adding 10 µl/well. The same procedure was repeated the day after. On the fourth day, cells were stimulated in duplicate for 24 h before incubating 20 µl of supernatant with Quanti-Blue as described above. The experiment was done in four biological replicates. T84 cells were seeded in 12-well plate in parallel of each experiment and transfected with the same protocol to verify *TLR2* and *TLR4* levels by qPCR following the method described below in two biological duplicates.

#### qPCR

Caco-2 cells were seeded for 21 days in a 6-well plate and medium was refreshed every 2–3 days. Medium was refreshed 6h before stimulation followed by total RNA extraction using RNeasy mini-Kit (Qiagen) according to the manufacturer’s recommendations. During the extraction process, lysates were treated with DNase I Rnase-Free DNase set (Qiagen). cDNA was synthesized from 2 μg of RNA using the High-Capacity cDNA Reverse Transcription Kit (Applied Biosystems) and 50 ng was used to conduct qPCRs on CFX96 (Biorad). The following Taqman Gene expression assay probes were used: Hs05465837_g1 *OCCLUDIN*, Hs00252666_s1 *CLAUDIN-2*, Hs01060665_g1 *ACTB*. *ACTINβ* was used for normalization. Samples were tested in three independent biological replicates. To evaluate siRNA efficiency, 50 ng were used to conduct qPCRs on CFX96 (Biorad) using SYBER Green (BioRad). Primers: *TLR2* 5’-TTA-TCC-AGC-ACA-CGA-ATA-CAC-AG-3’ and 5’-AGG-CAT-CTG-GTA-GAG-TCA-TCA-A-3’, *TLR4* 5’-AGA-CCT-GTC-CCT-GAA-CCC-TAT-3’ and 5’-CGA-TGG-ACT-TCT-AAA-CCA-GCC-A-3’, *GAPDH*: 5’-CCT-GCA-CCA-CCA-ACT-GCT-TA-3’ and 5’-GAC-TGT-GGT-CAT-GAG-TCC-TTC-C-3’.

#### TEER Measurement

Caco-2 were seeded at 25 × 10^5^ cells per well, in 12- wells Transwell plates with 0.4 μm pore polyester membrane insert (Corning 3460) and differentiated for 25 to 28 days. The medium was refreshed three times per week and changed before the experiment. Before reading, the plate was left to equilibrate 15min at room temperature, each read (R) was done at least in duplicate with the Millicell ERS ohmmeter (Millipore). The formula (Rsample − Rblank = Rcell layer)*area.cm² was applied to raw values, where Rblank is the resistance value from a non-inoculated well. In each experiment 5 µg of EVs were assessed in technical duplicate. The experiment was repeated three times independently.

#### Cytokines Measurement

HCT116, HT-29, T84 cells were seeded at 35 × 10^5^ cells per well in 12-wells plates and the next day medium was changed before adding drugs. After 24 h of incubation, the supernatant was centrifuged for 10 min at 300 g and stored at −80°C until use. Thp1 XBlue cells were seeded at 0.4 × 10^6^ on 12-wells plate the day prior stimulation. After 24 h of incubation, the supernatant was retrieved and centrifuged, as previously described. IL-8 was measured using IL-8 Human Uncoated ELISA Kit (Thermo Fisher, #88-8086) in half area plates (Corning 3695), following manufacturer instructions.

### Bacterial Culture

*Fusobacterium nucleatum* subsp *nucleatum* DSM 15643 - ATCC 23726 was cultured in DSMZ M104 medium depleted from meat extract and resazurin in Hungate tubes under CO_2_ or in bottles in an anaerobic cabinet (82% N_2_ and 18% CO_2_) for 24 h. Bacterial viability status was assessed before each EVs extraction using LIVE/DEAD BacLight Bacterial Viability Kit (Invitrogen). Contamination was regularly tested by microscopic inspection and aerobic growth test. Supernatant and non-inoculated control medium were obtained by centrifugation (4700 g, 20 min, 4°C and 10,000 *g*, 20 min, 4°C) and filtration on a 0.22 µM filter. Whole cell lysate of *F. nucleatum* was prepared as follows. Briefly, 2 ml of overnight culture was centrifuged at 11,000 rpm for 5 min and the pellet resuspended in 100 μl of RIPA buffer. Sample was incubated on ice for 5 min, followed by three cycles of 10 min boiling at 95°C and 10 min cooling at −20°. Sample was centrifuged and supernatant taken to perform protein quantification using Qubit.

### Extracellular Vesicles Purification and Characterization

EVs extraction was performed as described previously (25) with small modifications. Briefly, bacteria were grown as described above, centrifuged twice (4,700 *g*, 20 min, 4°C and 10,000 g, 20 min, 4°C). The supernatant was than filtered twice using a 0.22 μm filter before being concentrated using 100-kDA cutoff (Amicon) centrifugal filter units (Sigma Aldrich, # UFC910024). Amicon filters were centrifuged at 4,000 *g* (4°C) until all the supernatant went through the filter. Concentrated and EVs-enriched supernatant was finally rinsed twice using Optiprep diluent buffer (50 mM Hepes, 150 mM NaCl, and pH 6.8), and concentrated to a final volume of 500 μl.

**Figure 1:**
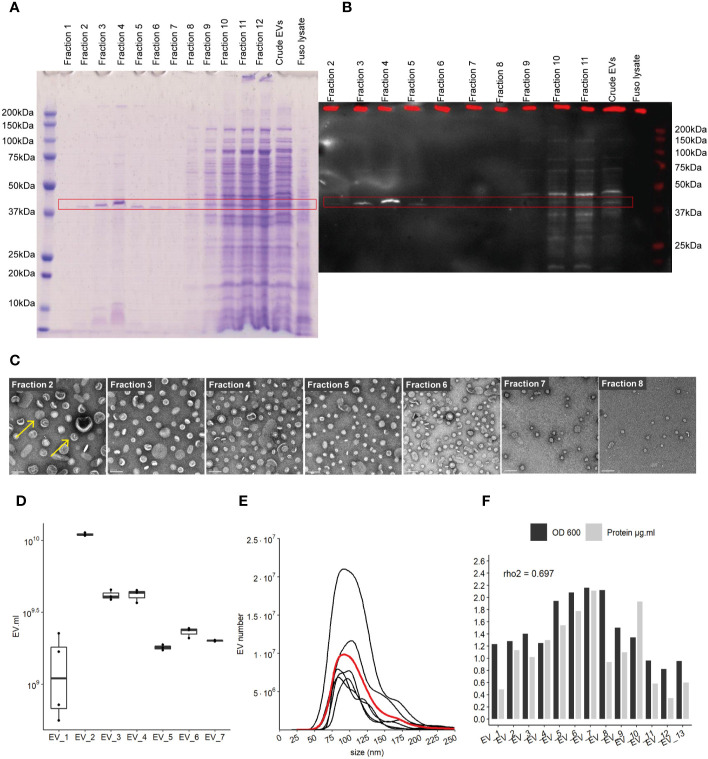
Characterization of EVs derived from *F. nucleatum*. **(A)** density gradient fractions of total protein profile stained with Coomassie Blue. **(B)** EVs fractions identified by far-western blot using a FomA specific biotinylated peptide derived from statherin protein, highlighted by the red rectangle. **(C)** EVs fractions 2 to 8 imaged by TEM; scale bar, 100 nm. Yellow arrows give examples of EVs structures **(D)** EVs count using NTA methods in 7 extraction batches, normalized by the volume of culture. **(E)** Size (nm) distribution of EVs in 7 distinct cultures and extractions, black lines represent individual EVs extraction, red line shows the mean distribution of 6 different purifications. **(F)** Protein concentration in each purification as determined by the BCA method and normalized by the initial volume of culture, biomass measured by optical density (OD) at 600 nm.

Pure EVs were isolated by iodixanol gradient (Optiprep, Sigma) using Beckman ultracentrifuge with a swinging bucket rotor (SW40 Ti) during 16 h at 100,000 *g* and 4°C. Therefore, continuous gradients were built with a bottom-up approach, loading vesicles in 2 ml bottom fraction containing 45% w/v iodixanol and overlaying successively 2 ml of 40%, 35%, 30%, 25%, and 20% w/v iodixanol fractions. After 16-h ultracentrifugation and deceleration with no break, 12 fractions of 1 ml were collected from the top low-density fractions to the bottom high-density fractions. 10 μg of whole cell lysate and 12.5 μl of each fraction was used to identify EV-containing fractions using 12% Bis-Tris SDS-page gel and subsequently staining with Imperial protein Coomassie stain (ThermoFisher Scientific) (see **Figure 1A**).

EVs-enriched fractions 2 to 8 were pooled and concentrated using 100-kDa cutoff (Amicon) centrifugal filter units and rinsed again with Optiprep diluent buffer. Finally, pure EVs were resuspended in Optiprep diluent buffer. Protein concentration of EV extractions was determined using Pierce BCA Protein Assay Kit (ThermoFisher Scientific), following supplier’s manual. EV extractions were ½ diluted and 10 µl/sample was used to perform BCA assay in microplates. The incubation time at 37°C was increased to 2 h, to net 562 nm measurement for each well and to lower both the minimum detection level of the reagent and the working range of the assay. An equivalent volume of non-inoculated medium was treated the exact using the exact same protocol than inoculated cultures. The resulting sample was used as “blank” in the experiments. Protein concentration was normalized on the initial volume of culture.

### Far-Western Blot

The protocol was adapted from (26). The statherin-derived peptide Biotin-Ahx-YQPVPE was synthesized by Proteogenix. After ultracentrifugation gradient, 25 μl of each fraction was resolved on a 12% Bis-Tris SDS-PAGE gel and the transfer was done by semi-dry conditions to a 0.45 µm PVDF membrane. The membrane was blocked overnight using 3% BSA and 1× PBS and incubated with 1 mg/ml peptide in 1× PBS with 0.5% Tween-20 for 1 h at RT. After three PBS-Tween washes, the FomA-peptide interaction was visualized by incubation with horseradish peroxidase (HRP)-conjugated streptavidin (Biolegend) diluted 1/2,000 in 3% BSA and 1× PBS. Image was acquired using either Pierce ECL substrate or SuperSignal West Femto Maximum Sensitivity Substrate (ThermoFisher Scientific) and Li-Cor ODYSSEY FC imager (Westburg)

### Microscopy

#### TEM

The presence of EVs after density gradient separation was confirmed by Transmission Electron Microscopy (TEM). For this purpose, EVs from 150 ml *F. nucleatum* culture grown in PYG medium were isolated as stated before. The 12 iodixanol fractions obtained after gradient ultracentrifugation were kept separated, washed with Optiprep diluent buffer and fixed with 4% paraformaldehyde during 10 min. A sample of the crude EVs preparation as before gradient separation was also fixed the same way. After fixation, each fraction was rinsed again with Optiprep diluent buffer and pelleted using a precooled Beckman ultracentrifuge with a 90Ti rotor during 2 h at 150,000 *g* at 4°C. The pellets were finally resuspended in each 100 µl Optiprep diluent buffer and 70 µl of each fraction was used to perform TEM. A 5 µl drop of each sample was deposited without prior dilution (except for crude EVs, diluted 10 times) on a freshly plasma cleaned (Fishione 1070) carbon-coated Electron Microscopy (EM) copper grid (430 mesh square grid, Euromedex CF300-CU-050). After 1 min of application, the sample was washed with water three times to remove any buffer salts, which would react with uranyl acetate used for negative staining. To perform negative staining, the grid was washed twice with 50 µl of uranyl acetate solution (2%) and the excess of stain was blotted out using a Whatman filter. The grids were kept in a dry, dark, dust-free environment until observation with the electron microscope. For sample observation, the EM grids were mounted on to a room temperature equilibrated holder and subsequently introduced into a FEI Tecnai 20 transmission electron microscope (FEI Eindhoven Holland) operating at a voltage of 200 kV. Images (2,048 pixels × 2,048 pixels) were acquired using a US1000 camera (Gatan) at 29,000 X for the 100-nm scale bar pictures and 5,000 X for the 500-nm scale bar pictures.

#### Fluorescent Microscopy

EVs were fluorescently labeled with FITC as described previously (27). Briefly, EVs were mixed 1:1 with 1 mg/ml FITC (Sigma-Aldrich) in 50 mM Na2CO3 and 100 mM NaCl, pH 9.2 and incubated for 1 h at 25°C. Labeled EVs were washed three to four times with PBS using 100-kDa cutoff Amicon centrifugal filter units (Sigma Aldrich, # UFC510024). Human cells were washed twice with PBS and fixed with 3.7% PFA. Cover slips were incubated with or without Wheat Germ Agglutinin Fluor 647 (Life Tech) and were mounted using Fluoroshield mounting medium containing DAPI (Sigma # F6057). Image were acquired with an Olympus IX83 microscope, 100x objective. Images were treated by applying the same level and window settings for all channels and pictures with Fiji ImageJ.

### EVs Counting and Profiling

EVs were counted using NanoSight NS300 (Malvern Panalytical) using level setting at 15 with a dilution of 1/5,000 or 1/10,000 in PBS, depending of the samples concentration after testing the machine calibration with beads. Analysis settings of blur and Jump were in automatic mode. EVs concentration was normalized on the initial volume of culture.

### LPS Extraction

LPS was purified using the hot phenol–water method (28) and further purification was performed using a modified phenol re-extraction protocol (29). LPS was isolated from 1.5 g of bacterial pellet, obtained from 500 ml *F. nucleatum* culture grown in PYG medium. Bacterial pellet was resuspended in 5 ml SDS buffer and the volume of further reagents was increased accordingly. Both, aqueous and phenolic phases were recovered. The aqueous phases were pooled together and adjusted to 75% ethanol and 30 mM sodium acetate final concentration and allowed to precipitate at −20°C for 1 h. The precipitates were centrifuged at 4°C for 10 min at 10,000 *g*, washed in 1 ml of cold 100% ethanol, and air-dried. The weight of the dried LPS pellet was balanced.

The phenolic phase was mixed with 9 volume of acetone and allowed for precipitation at −20°C. The precipitate was dialyzed against distilled water using 10K kDa Slide-A-Lyzer G2 Dialysis Cassettes (Life Technologies), following supplier manual. Briefly, the phenolic precipitate was mixed with 2 volumes of distilled water and filled into hydrated cassettes. Cassettes were dialyzed against distilled water for 2 h, water was exchanged and dialyzed overnight. Finally, cassettes were dialyzed again for 2 h against fresh distilled water. Samples were collected and centrifuged for 30 min at 10,000 *g* (4°C), washed in 1 ml of cold 100% ethanol, and dried in vacuum globe. The weight of the dried LPS pellet was balanced. LPS concentration of bacterial pellet and EV extractions were quantified using an ELISA-based endotoxin detection assay (Endolisa, Hyglos), following manufacturer instructions. Furthermore, total carbohydrates in EV extracts were quantified using Total Carbohydrate Assay Kit (Sigma-Aldrich), using additional glucose standards and following supplier’s manual. LPS was visualized with 50 µg of aqueous phase LPS, 50 µg of phenolic phase LPS, 50 µg of mixed aqueous and phenolic phase LPS were visualized on a gel. *E.coli* Ultrapure LPS (50 µg) was used as a positive control. Briefly, samples were prepared with Laemmli sample buffer, heated for 10 min at 100°C and separated on a 12.5% Bis-Tris precast gel. Silver staining of the gel was performed using a corresponding kit (SilverQuest, ThermoFisher), according to the manufacturer’s instructions. LPS concentration was normalized on the initial volume of culture.

### Quenching Assay

T84 cells were seeded at 30,000 cells per well one day prior experiment on a 96-wellsblack plate. *F. nucleatum* EVs and the same volume of blank of labeled were with FITC for 1 h at 25°C, washed three times on Amicon and kept at -20°C, as previously in the microscopy method. On the day of experiment the FITC-labeled EVs and blank were incubated on the cells for 4 h at 37°C, before assessing the fluorescence at 485 and 528 nm, bandpass 20 nm, sensitivity 80, using Synergyx Mx plate reader (Biotek Instruments). Cells were washed with PBS and external FITC fluorescence was quenched with 0.2% Trypan blue. Finally cells were washed two times with PBS. Fluorescence was read at each step using the same settings, adapting published protocols (30, 31). The experiment was done in four independent biological replicates.

### EVs Incubation With FomA Peptide

To reduce FomA, affinity to human cell receptors, 2 µg of *F. nucleatum* EVs and the same volume of blank were mixed with 100 µl of 2 mg.ml^−1^ or 4 mg.ml^−1^ of statherin-derived peptide (described in the Far western blot method) in PBS with 0.05% Tween-20 and incubated for 2 h at 37°C. EVs were washed two times with PBS using 100-kDa cutoff Amicon centrifugal filter units and washed for a third time with DMEM-F12 medium. Three independent EVs preparation has been done with 2 mg.ml^−1^ of peptide and 2 preparations with 4 mg.ml^−1^ of peptide.

### Statistical Analysis and Graphics

Statistical analysis was done using non-parametric Wilcoxon rank test and single data points were represented using ggplot boxplot resenting median, first and third quartiles. Data come from at least three independent biological experimentations.

Data were analyzed using R and RStudio software. Graphics and statistical analysis were produced with ggplot2, ggsci, gridExtra, ggsignif, and ggpubr packages. Statistical test used was Wilcoxon rank test otherwise stated. Fiji (ImageJ 1.52p) was used with the OlympusViewer plugin to analyse fluorescence microscopy images.

## Results

### Characterization of *F. nucleatum* EVs

*F. nucleatum* subsp *nucleatum*, type strain, was cultured in a meat extract depleted medium to reduce the potential presence of EVs derived from the medium, before harvesting the supernatant (32). To recover highly purified EVs, they were extracted by density gradient ultracentrifugation (25, 33). Concentrated EVs before, referred to as “crude”, and after density gradient ultracentrifugation were evaluated on a SDS-PAGE gel. The total protein content of each EV’s gradient fractions was assessed in SDS-PAGE gel, in which the strongest band was visible around 41 kDa in fractions 2 to 8 (**Figure 1A**). No band was visible on control extraction gel (“Blank”) which served as the negative control in this study (**Figure 1SupA**). We next investigated the presence of the OMP FomA using a biotinylated peptide derived from the salivary statherin protein, using the far-western blot technique (**Figure 1B** and **Figures 1SupB, C**). This peptide has described previously to directly bind to FomA (17). FomA, between 37 and 50 kDa, was mainly present in EVs contained in fractions 3 to 5, with a marked signal in fraction 4. The assay may not be sensitive enough to detect lower concentration in other fractions. Each fraction was further characterized by transmission electronic microscopy (TEM) to confirm the presence of EVs (**Figure 1C** and **Figures 1SupD, E**). In TEM, EVs were mostly present in fractions 2 to 8, which is in accordance with our SDS-PAGE assay results. On the contrary, fractions 1 as well as 10 to 12 displayed very few particles despite containing high protein levels. These results suggested that proteins found in the denser fractions 10 to 12 of the gel were contaminants rather than proteins contained within EVs. It is worth noting that FomA, as well as other contaminant proteins and vesicles were found in the “crude” EV preparations before density gradient separation by far-western blot and TEM observation. Because fractions 2 to 8 were enriched in purified EVs, these fractions were pooled together for further study and referred to as “EVs” in the rest of the study. Our results showed that density-gradient ultracentrifugation is an efficient method to separate non-vesicles from vesicles and yields high EVs purity. Interestingly, the selected EV fractions showed a different profile in terms of protein content compared to the denser and non-EVs fractions, with apparently less protein diversity in EVs.

Despite differences in the optical density (OD) of each *F. nucleatum* culture, the quantities of EVs were comparable across several extraction batches measured by Nanoparticle Tracking Analysis method (NTA). EV concentrations ranged from 2.10e+9 to 5.51e+10 EVs per ml of culture, with a mean of 1.75E+10 (SD 1.77e+10) and median of 1.33e+10 (**Figure 1D**). The EV population median size was found to be between 104 and 105 nm (**Figure 1E**). Minor populations of larger EVs were visible in several extractions with sizes around 125 and 175 nm. The protein content varied between batches and was correlated with the relative biomass measured by the OD (rho2 = 0.69) (**Figure 1F**). We also demonstrated that FomA was abundant in EVs similarly to the outer membrane of the bacterial cell (34).

### EVs Induce Different NF-κB Responses in the T84 Intestinal Epithelial Cells

**Figure 2:**
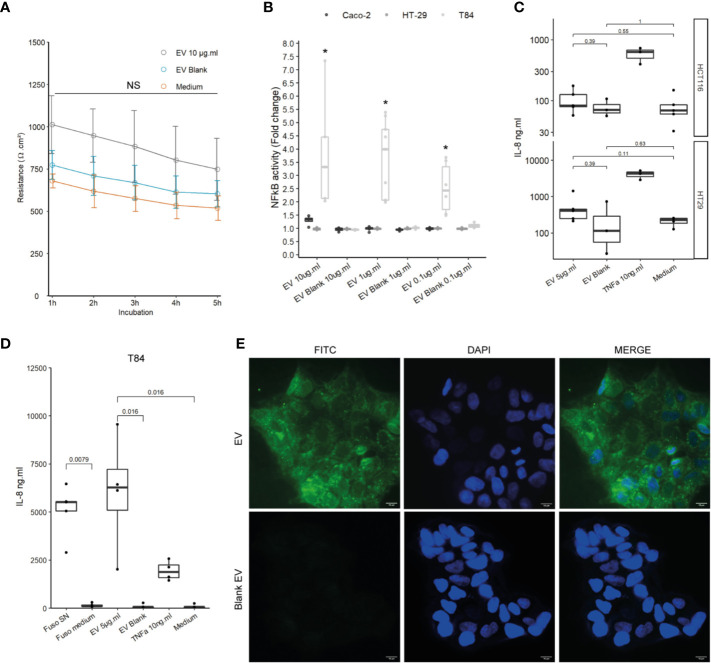
Impact of EVs on intestinal epithelial cells and innate immunity. **(A)** Epithelial resistance monitored in Caco-2 differentiated cells exposed to 10 µg.ml^−1^ EV for 5 h or Blank EV (Ω.cm²). Data are represented as the average of three experiments ± SD. Exact p-value of EVs vs Blank EV were 1 h: 0.110; 2 h: 0.093; 3 h: 0.104; 4 h: 0.188; 5 h: 0.199 (T test). **(B)** NF-κB activation level in Caco-2, T84 and HT-29 NF-κB reporter cell lines after incubation of 24 h with different EVs concentrations. Caco-2 and HT-29 p-values were not significant. T84 p-value from left to right: 0.0357, 0.0090, and 0.0095. Data are expressed as median ± quartiles of fold change toward unstimulated cells. **(C)** Secreted IL-8 measured by ELISA in HCTT16, HT-29 stimulated with 5µg.ml^−1^ EVs, Blank EV or TNF-α 10ng.ml^−1^ for 24 h (N = 3). **(D)** Secreted IL-8 measured by ELISA in T84 cells stimulated with *F. nucleatum* supernatant, control medium, 5 µg.ml^−1^ EVs, Blank EV or TNF-α 10 ng.ml^−1^ for 24 h (N = 4). **(E)** (Upper left panel) 5 µg of FITC-labeled EVs or (Lower left panel) 5 µg of Blank EVs, were incubated on T84 cells for 3 h, 100X, FITC (Green), nucleus DAPI (Blue); scale bar, 10µm.

We next studied the effects of *F. nucleatum* EVs on host IECs, which are host interface between the host’s inner environment and the intestinal microbes. We first tested whether exposure of IECs to *F. nucleatum* EVs affects the epithelial barrier function. In Caco-2 differentiated cells, exposure to EVs for 5h had no significant impact on epithelial resistance, suggesting that EVs did not increase epithelial permeability (**Figure 2A**). Furthermore, EVs had no negative impact on *Occludin* and *Claudin*-2 mRNA levels, two important proteins that maintain tight junctions (**Figure 2SupA**). Because Caco-2 cells are known to be produce a low NF-κB response to microbial stimulation, other IEC lines were tested (35, 36). Thereafter, we tested whether exposure to EVs resulted in NF-κB activation in different IEC lines, a central transcription factor which integrates innate immunity responses. After 24 h of exposure, EVs induced activation of NF-κB in T84 cells while Caco-2, HT-29 cells showed no or low NF-κB response (**Figure 2B**). We furthermore observed that EVs provoked a significant IL-8 secretion in T84 but not in HCTT16 and HT-29 cells (**Figures 2C, D**). Exposure of T84 cells to EVs produced cytokine release that was of the same order of magnitude as *F. nucleatum* supernatant. We next examined if EVs were also able to adhere to T84 by monitoring fluorescence of FITC-labeled vesicles after washings. Indeed, *F. nucleatum* expresses several adhesion proteins, such as FadA, allowing bacterial adhesion and invasion different human cell types including IECs. FITC labeled-EVs were visible on microscopy, with a diffuse foci pattern on T84 cells (**Figure 2E** and **Figure 2SupB**). We then tested if after binding, EVs were internalized by T84 cells. However, after 4-h incubation with FITC-EVs, we observed that fluorescence was lost after Trypan blue quenching suggesting that EVs were not massively internalized into cells (**Figure 2SupC**).

### *F. nucleatum* EVs Are Weak Activators of TLR4 in IECs

**Figure 3:**
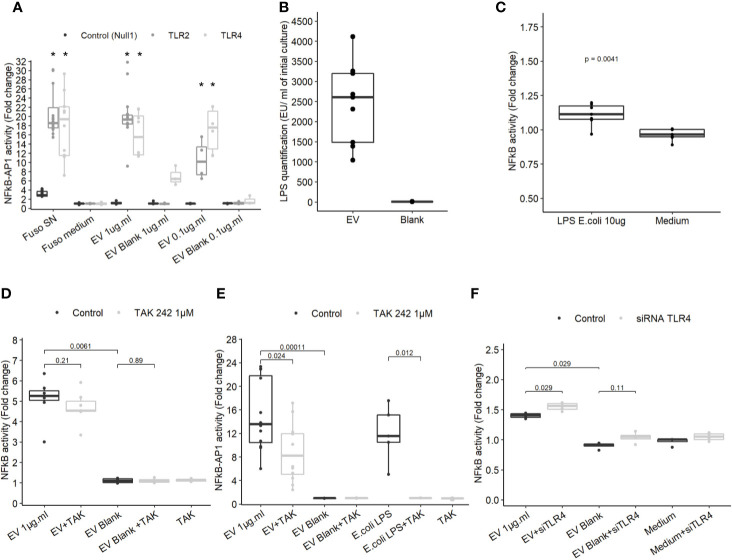
TLR4 activation is dispensable in NF-κB activation by EVs. **(A)** NF-κB-AP1 activation after EVs exposure for 24 h on HEK-293 Null1, TLR2 and TLR4 reporter cell-lines (N ≥ 3), * indicate p-value <0.05. **(B)** LPS concentration (EU.ml) normalized by the volume of culture (Endotoxin unit). **(C)** NF-κB activation on T84 cells upon exposure to 10 µg.ml^−1^ of ultrapure *E. coli* 055:B5 LPS for 24 h (N = 7). **(D)** NF-κB activation by 1µg.ml^−1^ EV for 24 h in T84 cells with or without TLR4 inhibitor pre-treatment (TAK-242 1µM) (N ≥ 4). **(E)** NF-κB-AP1 activation by 1 µg.ml^−1^ EV 1 µg.ml or 100 ng.ml^−1^ of ultrapure *E.coli* LPS for 24 h in Thp1 XBlue cells with or without TLR4 inhibitor pre-treatment (1 µM TAK-242) (N ≥ 4). **(F)** NF-κB activation following 1µg.ml^−1^ EV exposition for 24 h in T84 cells treated with siTLR4 or control siRNA (N = 4). Data from **Figures 4A, C–F** are expressed as median ± quartiles of fold-change toward unstimulated cells.

To determine which molecules trigger the NF-κB pathway, EVs activity was tested on different HEK cell lines over-expressing specific innate immune receptors. EVs did not induce activation in HEK Null1 cell line expressing TLR3, TLR5 and NOD1 and neither in HEK cell overexpressing NOD1 and NOD2 (**Figure 3A** and **Figure 3ASup**). In contrast, EV activated TLR2 and TLR4 receptors in a dose dependent manner (**Figure 3A**). TLR2 is activated by various and heterogeneous ligands (lipoproteins, proteins, polysaccharide, etc.) while TLR4 signaling is triggered by lipopolysaccharide (LPS) (37). T84 cells express TLR4 but they have a weak CD14 expression, facilitating LPS binding, and thus were weakly activated by LPS. Expression of both TLR2 and TLR4 by T84 cells may explain why the cell line is more sensitive to *F. nucleatum* EVs than other IECs (38, 39). Thus, T84 IECs were used in the following experiments to study the molecular mechanisms underlying NF-κB activation in more detail. The *F. nucleatum* strain used in this study harbors a smooth type LPS previously characterized (40). The LPS concentrations on EVs were quantified in different extractions (**Figure 3B**). The LPS concentrations were variable between culture batches, with a high LPS content detected along with high protein concentration. The LPS concentrations in EVs ranged from 1041 EU.ml^−1^ to 4112.4 EU.ml^−1^ corresponding approximately to 104.1 ng to 411.24 ng.ml^−1^ LPS from *E. coli* (10 EU.ml^−1^ = 1.0 ng.ml^−1^). A carbohydrate-based LPS quantification was tested but due to low sensitivity, the results could not be interpreted. We next tested which LPS concentration was required for NF-κB activation in T84 cells. When cells were stimulated with 10 µg.ml^−1^ of *E. coli* ultrapure LPS, a low NF-κB activation was observed (**Figure 3C**), suggesting that TLR4 may not be key in the EVs-dependent activation. To further rule out the LPS-TLR4 signaling pathway’s implication, T84 cells were pre-treated with a TLR4 specific inhibitor (TAK-242). We observed that TLR4 inhibition did not prevent EVs activity in T84 cells (**Figure 3D**). TLR4 inhibition efficiency was tested by pre-treating THP1 monocytes with TAK-242 before incubation with EVs or *E. coli* purified LPS. In THP1 cells, TLR4 inhibition significantly decreased cell response, indicating that TAK-242 was effective in decrease TLR4 signaling after LPS stimulation (**Figure 3E**). TLR4-independent activation was further confirmed in T84 cells using a TLR4 siRNA. The TLR4 level had no impact on the NF-κB response induced by *F. nucleatum* EVs (**Figure 3F** and **Figure 3SupB**). Taken together, our results suggested that TLR4 and the LPS contained within *F. nucleatum*-derived EVs were not the main responsible of NF-κB activation in T84 cells. The *E. coli* LPS structure impacts EVs endocytosis and fusion to host cells (41). Therefore, we investigated if the weak activation of TLR4 by EVs, could be due to an abnormal LPS. However, the LPS extracted from the whole bacterium revealed the presence of the full LPS structure as described previously (**Figure 3SupC**) (40). The LPS structure contained on EVs could not be resolved because the large EV quantity necessary could not be obtained.

### NF-κB Activation by *F. nucleatum* EVs Is TLR2- and FomA-Dependent in IECs

**Figure 4:**
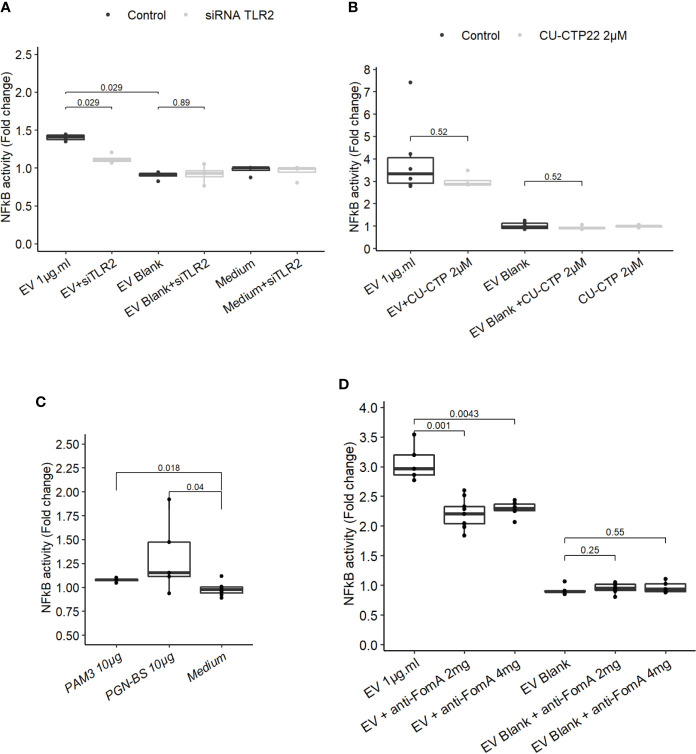
FomA contained in EVs was involved in NF-κB activation and was TLR2-dependent. **(A)** NF-κB activation by 1 µg.ml^−1^ EV for 24 h in T84 cells with siTLR4 treatment or control siRNA. Controls used in this figure are shared with the **Figure 4F** as all siRNA experiments were done together. **(B)** NF-κB activation by 1 µg.ml^−1^ EVs for 24 h in T84 cells pre-treated 2 h before with TLR2-TLR1 inhibitor (2 µM CU CTP22). **(C)** Stimulation of NF-κB with 10µg.ml^−1^ PAM3CSK4 (TLR2/TLR1 synthetic ligand) or 10 µg.ml^−1^ PGN-BS (*B. subtilis* peptidoglycan) in T84 after 24 h. **(D)** NF-κB activation by 1 µg.ml^−1^ EVs pre-incubated with 2 mg.ml^−1^ or 4 mg.ml^−1^ of FomA specific peptide compared to control EVs, incubated for 24 h in T84 cells (N ≥ 4). Data are expressed as median ± quartiles of fold-change toward unstimulated cells.

Because our results indicated that TLR4 was dispensable to promote EV-mediated NF-κB activation, we next investigated whether TLR2 was involved not only in HEK cells overexpressing the receptor but also in IEC immune-modulations. When TLR2 level was knockdown by treatments with siRNAs in T84 cells, the NF-κB activation following EVs exposure was decreased (**Figure 4A** and **Figure 3SupB**). This result indicated that NF-κB stimulation by EVs was dependent on the TLR2 expression level and therefore its signaling. We noticed that activity of EVs was reduced after two rounds of siRNA transfections compared to no transfection. To decipher further the mechanisms involved, T84 cells were pre-treated with a TLR2-TLR1 heterodimer specific inhibitor (CU-CTP22) (42). Yet, the TLR2-TLR1 inhibition had no impact on EVs activity (**Figure 4B**). In line with this, we found that the synthetic TLR2-TLR1 ligand (PAM3SCK4) did not trigger the NF-κB pathway, while another TLR2 activator, the *B. subtilis* peptidoglycan was able to induce a stronger response (PGN-BS) (**Figure 4C**). However, the response to *B. subtilis* peptidoglycan produced a reduced response compared to EVs (**Figures 4B, C**). Because the major OMP FomA has been shown to directly activate TLR2, we hypothesized that this protein may also be involved in EVs activity. To test this hypothesis, we used the statherin-derived peptide which binds to FomA (“anti-FomA”) (**Figure 1B**) and, thus, prevents binding to cells receptors. EVs and Blank were pre-incubated with the statherin peptide for 2 h and washed before testing their activity on T84 cells (**Figure 4D**). EVs treated with the competitive inhibitor peptide revealed a reduced NF-κB activation compared to non-treated EVs, demonstrating that FomA was involved in cells innate immune response.

### Host Cells Dynamin Is Required for NF-κB Activation by EVs

**Figure 5:**
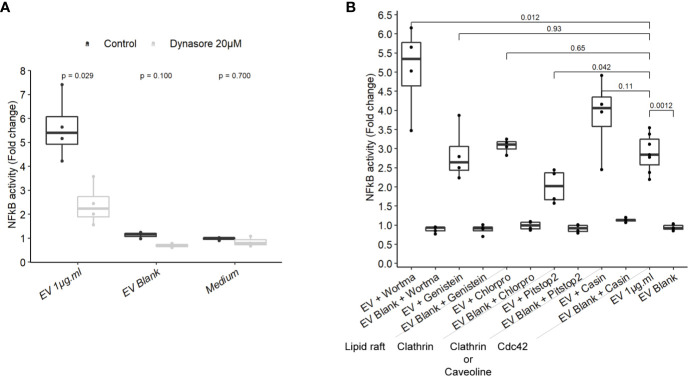
NF-κB activation by EVs relied on a dynamin-dependant endocytosis. **(A)** NF-κB activation by 1 µg.ml^−1^ EVs for 24 h in T84 cells pre-treated 2 h before with 20µM dynasore (N = 3). **(B)** NF-κB activation by 1 µg.ml^−1^ EV for 24 h in T84 cells pre-treated 2 h before with endocytosis inhibitors wortmannin 20 µM, genistein 50 µM, chlorpromazine 5 µM, pitstop2 20 µM, or Cdc42 inhibitor casin 10 µM (N ≥ 3). Data are expressed as median ± quartiles of fold change toward unstimulated cells.

To gain more insight on EV mechanisms in stimulating innate immunity, we examined the role of different endocytosis routes. Dynamin-mediated membrane trafficking was blocked by pre-incubating T84 cells with dynasore, a GTPase dynamin inhibitor. In this setting, NF-κB activity was strongly reduced, suggesting that EV-mediated signaling necessitated dynamin (**Figure 5A**). Dynamin mediates budding of cell vesicles coated both with clathrin and caveolin. Inhibition of clathrin-coated pits assembly at cell surface with chlorpromazine had no impact on the activity of EVs (**Figure 5B**). In contrast, inhibition of both clathrin-dependent and clathrin-independent endocytosis by a broad inhibitor (pitstop2), slightly reduced NF-κB activation (43). Next, we evaluated if the remaining EVs activity during the blockage of dynamin-mediated endocytosis could be explained by a lipid-raft uptake, a clathrin-independent pathway. Lipid-raft mediated uptake blockage (genistein) did not affect NF-κB activation by EVs. In addition, pre-treatment with PI3K inhibitor (wortmannin), which also prevents lipid raft-dependent uptake, did not reduce the stimulation of cell but rather increased it. Lastly, we looked at the role of Cdc42, belonging to the Rho GTPases family which plays a central role in regulating endocytosis of proteins attached to the membrane by glycosylphosphatidylinositol anchor (GPI). Pre-treatment with Cdc42 inhibitor (casin) did not lead to reduced EVs stimulation. Altogether, our data indicated that EV-mediated signaling required the dynamin-mediated budding of vesicles while lipid-raft-mediated cellular uptake was dispensable. This suggested that EVs signaling necessitates clathrin-independent endocytosis.

## Discussion

In this work, we aimed first at isolating EVs derived from *F. nucleatum* to characterize their biophysical parameters. To achieve the first objective *F. nucleatum* was cultured in a depleted medium to reduce the presence of external contaminant EVs and a density gradient ultracentrifugation step was performed to obtain a higher degree of purification (25, 33). The purity and specificity of EVs were confirmed by protein staining and electronic microscopy. The quantities of EVs measured by the nanoparticle tracking method was about 10^10^ EVs per ml of original culture. The fluctuations of the EV yield in different batches of culture were correlated to the relative biomass evaluated by OD and protein content. Our data suggest that despite a controlled culture environment, unidentified physiological parameters regulate the amount of EVs produced as well as their relative protein content. EV formation and cargo is triggered by many factors: growth conditions, iron or oxygen levels, SOS response or cell envelope stress (44, 45). We found that the major EVs population median size was around 100 nm with minor populations of larger size. *F. nucleatum-*derived EVs size is in line with those reported on diverse other bacterial species (44). We were able to show that FomA porin was present in EV-enriched density-gradient fractions. This is interesting as FomA has been shown to be one of the main constituents of the *F. nucleatum* whole bacterial outer cell membrane. FomA has a strong affinity to the human statherin protein and immunoglobulins Fc receptors (17, 34, 46). We observed a shift in the FomA band in far-western blot, particularly pronounced in the fraction 4. This was present in all the methods used (blot, Sliver stain, Coomassie) and across all EV extractions batches. Our main hypothesis is that the FomA may be linked to remaining lipids despite the denaturing conditions explaining why the band is bent. We previously observed the same phenomena in past studies with another membrane protein.

Secondly, we evidenced that EVs released by *F. nucleatum* induced NF-κB activation in IECs. We found that EVs promote NF-κB transcriptional activities by activating both TLR2 and TLR4 in HEK cells. We hypothesized that the differential impact of EVs on IECs was driven by TLR4 and/or TLR2 expression levels. Previous publications have shown that TLR4 and TLR2 mRNAs were not detectable in HCTT16 cells. In contrast, HT-29 and T84 cells both express TLR4 but they do not express CD14, facilitating LPS binding, and thus they are weakly activated by LPS. Concerning TLR2, HT-29 and T84 cells do not have the same expression profiles, T84 cells express TLR2 but HT-29 do not (38, 39). TLR2 expression in T84 cells reflect the profiles observed in the proximal and distal colonic epithelial cells assess in human primary tissues. The expression level of TLR4 in the human colon is apparently more variable between studies compared to TLR2 (47–49).

Our results indicated that EVs have immunomodulatory properties comparable those shown with the whole bacterium in HEK cells (50, 51). EVs from other bacterial species have been shown to be rich in LPS and to induce TLR4 and Caspases-11 activation after internalization (45, 52, 53). Our results indicated that *F. nucleatum*-derived EVs were also rich in LPS. However, TLR4 was dispensable in mediating NF-κB activation in IECs. Moreover, a strongly immunogenic LPS derived from *E. coli* did not mimic EV activation. The inflammation level induced by *F. nucleatum* LPS remains undetermined. Early publications on *F. nucleatum* LPS have described pronounced inflammatory properties (54–56). However, when highly purified LPS has been tested, in particular with no peptidoglycan contamination, its immunogenicity was reduced compared to LPS derived from *E. coli* O111:B4 or other proteins derived from *F. nucleatum* (34, 40). Moreover, different studies challenging HEK cells and murine macrophages with *F. nucleatum* bacteria showed that TLR4 has a minor role in cellular inflammatory responses (50, 51). We thus analyzed the *F. nucleatum* LPS, using a specific extraction method to recover full length LPS from cells. We confirmed that the type strain expresses smooth type LPS in our experimental settings, as described previously when checking both aqueous and phenolic extraction phases (40). Whether the LPS structure contained in EVs is similar to LPS structure from the whole cell remains an opened question. Apart from LPS direct interaction with TLR4, another explanation for a weak immune response is that LPS presence on the EV surface impacts vesicle interaction with host cell membrane. Indeed, LPS structure and O-antigen presence particularly, have been demonstrated to mediate EV interactions with human cell membranes by favoring internalization and fusion (41, 45). Thereby, the LPS structure of *E. coli* pathogenic isolates plays an important role in determining EV route into host cells and immune-stimulation: EVs derived from commensal *E. coli* are much less internalized compared to EVs produced by HEPEC *E. coli.* On the other hand, the IEC lines used in the present study expressed low level of LPS receptors and co-receptors MD2 or CD14, which might also contribute to explain our findings.

Our experiments showed that *F. nucleatum* EVs response in IECs is TLR2-dependent. The response was not impaired by TLR2-TLR1 inhibition, suggesting that another TLR2 dimer, homodimer, or heterodimer could be involved (57, 58). Another possibility is that the chemical inhibitor was not stable enough to prevent the TLR2-TLR1 activation (59). Because HEK cells do not express TLR1 or TLR6, we speculate that a TLR2-TLR2 homodimer was involved. Different TLR2 ligands had weak activities in T84 cells which opened the possibility that the innate immunity activation could rely on FomA porin. We have demonstrated that FomA was present on *F. nucleatum* EVs, and importantly, this porin has been shown previously to be a TLR2 agonist (18). Pre-treatment of EVs with anti-FomA binding peptide resulted in a reduction of EVs activity in IECs, manifesting that FomA presence on EVs was involved in NF-κB activation probably *via* TLR2. We do not know if the partial observed effect was due to a limited peptide affinity toward FomA and/or to a limited coverage of the TLR2 binding site. In this case, mutation of FomA in *F. nucleatum* would be necessary to fully confirm this mechanism.

Finally, we established that EVs activation was dependent of the endocytosis driven by the large GTPase dynamin, similar to results reported using *E. coli* and *T. vaginalis* EVs (41, 53, 60). Furthermore, our data indicate that the clathrin-dependent endocytosis as well as the lipid-raft-mediated cellular uptake were dispensable. Lipid raft is a frequent entry mode at least for gram negative-derived EVs such as *P. gingivalis* or *H. influenza* (23). The slight inhibition of NF-κB activation observed with Pitstop2 inhibitor also suggests that EVs signaling relied on clathrin-independent endocytosis. However, as this inhibitor has several cellular targets, resolution of the specific pathway would require further experimental validations (43, 61).

Our data suggest that *F. nucleatum* EVs induce mostly a TLR2-dependent stimulation. Under homeostatic conditions, TLR2 stimulation by *F. nucleatum* may not necessary result in a pathological inflammatory response. This is supported by studies showing that in mice, activation of TLR4 and TLR2 by *F. nucleatum* bacteria actually suppresses inflammation and promotes regulatory T (Treg) cells (19). More generally, the pathogenicity of one species does not symmetrically correspond to EVs pathogenicity derived from the same species. Hence, the acute pathogen *V. cholerae* has been shown to produce EVs with low inflammatory potential (62). Our results show that EVs do not alter the intestinal epithelial barrier but stimulate TLR2. These findings are in line with previous reports indicating that TLR2 has an important role in maintaining intestinal homeostasis and protection of the mucosa from injury (63, 64). Stimulation of TLR2 by the gut microbiota or the polysaccharide A (PSA) from *B. fragilis*, have been shown to have protective role in the gut, reducing inflammation by increasing IL-10 production by dendritic and B cells and promoting Treg proliferation (65–67). Furthermore, *B. fragilis* EVs contain PSA, which is sufficient to promote tolerogenic dendritic cells and protect from inflammatory disease (68).

Even though the observed EV-based stimulation of TLR2 may indicate possible beneficial effects, human *Tlr2* polymorphisms have complex roles in several diseases, including rheumatoid arthritis, type I diabetes or colorectal cancer (69). In the gut, specific *Tlr2* polymorphisms have protective effects while others are associated with an increased risk of colorectal cancer depending on the populations (70–73). Interestingly, *Tlr2* but not *Tlr4* expression is increased in cancerous tissues compared to non-cancerous adjacent tissues (47, 74). Our work indicates that if TLR2 activation by EVs do not disrupt healthy intestinal epithelium, TLR2 activation can have a deleterious impact in the context of colorectal cancer. Therefore, besides Fap2 and FadA, it would be interesting to test the role of EVs and FomA in particular in the colorectal tumorigenesis.

Very little is known on how EVs, derived from the gut microbiota, impact human gut physiology locally or distantly, and whether EVs can cross the epithelial barrier. Among the major open questions, the range of EV concentrations along the gastro-intestinal tract and how they diffuse from the lumen to the mucosa are probably key. All these factors are necessary to better understand the roles of EVs from species with versatile pathogenic potential such as *F. nucleatum* or *E. coli*, in the global mechanism by which the gut microbiota modulate gut homeostasis.

## Data Availability Statement

The raw data supporting the conclusions of this article will be made available by the authors, without undue reservation.

## Author Contributions

CM-G and AM conceived and designed the experiments. CM-G, AM, and JH performed the experiments. CM-G and AM analyzed the data. CM-G, AM, and JH wrote the paper. CM-G, AM, JH, and PW edited and revised the manuscript. All authors contributed to the article and approved the submitted version.

## Funding

This work was supported by the Luxembourg National Research Fund (FNR) Microcancer grant (CORE/15/BM/10404093) and Bilateral FNR grant (AFR Bilateral/11228353-4).

## Conflict of Interest

The authors declare that the research was conducted in the absence of any commercial or financial relationships that could be construed as a potential conflict of interest.

## Supplementary Material

Supplementary Figure 1
*F. nucleatum* EVs purification and TEM. **(A)** density gradient fractions total proteins profile of EVs and corresponding blank, stained with Coomassie Blue. **(B)** Second EVs fractions identified by far-western blot using a FomA specific biotinylated peptide using classical revelation method. **(C)** Second EVs fractions identified by far-western blot using a FomA specific biotinylated peptide using Femto sensitive revelation method. **(D)** density gradient fractions total proteins profile of EVs batch corresponding to far-western blot presented in 1SupB and C, stained with Coomassie Blue **(E)** EVs fractions 1 to 12 imaged by TEM and crude extract (0.1×); size bar, 100nm.

Supplementary Figure 2
**(A)** Caco-2 differentiated cells were incubated with 5µg of EVs or Blank for 6h before assessing *Occludin* and *Claudin-2* mRNA levels, normalized on *Actin* (N = 3). **(B)** FITC-labeled EVs were incubated on HT29 for 6 h, 40X, FITC (Green), nucleus DAPI (Blue). **(C)** Incubation of 5 µg FITC labeled EVs or Blank for 4 h on T84. Fluorescence level was read after incubation, after PBS washes and after quenching external fluorescence with 0.2% Trypan blue (N = 4).

Supplementary Figure 3
**(A)** Activation of NF-κB-AP1 following EVs exposure of 24 h on HEK-293 NOD1 and NOD2 reporter cell-lines (N ≥ 3) **(B)** Efficiency of siRNA targeting *TLR2* and *TLR4* respectively assessed by RT-PCR normalized to *GAPDH* (N = 2). **(C)** *F. nucleatum* LPS extraction on a 12.5% SDS-PAGE gel visualized with Silver stain. Line 1: ladder, Line 2: 50 µg aqueous phase LPS, Line 3: 50 µg phenolic phase LPS, Line 4: 50 µg of mixed (1:1) aqueous and phenolic phase LPS, Line 5: Control (50 µg of *E. coli* Ultrapure LPS).
