# Supplementary figures and images for: Fusobacterium nucleatum Extracellular Vesicles Modulate Gut Epithelial Cell Innate Immunity via FomA and TLR2
Source: Front Immunol. 2020 Dec 21;11:583644. doi: 10.3389/fimmu.2020.583644 (PMC7779620; doi:10.3389/fimmu.2020.583644)

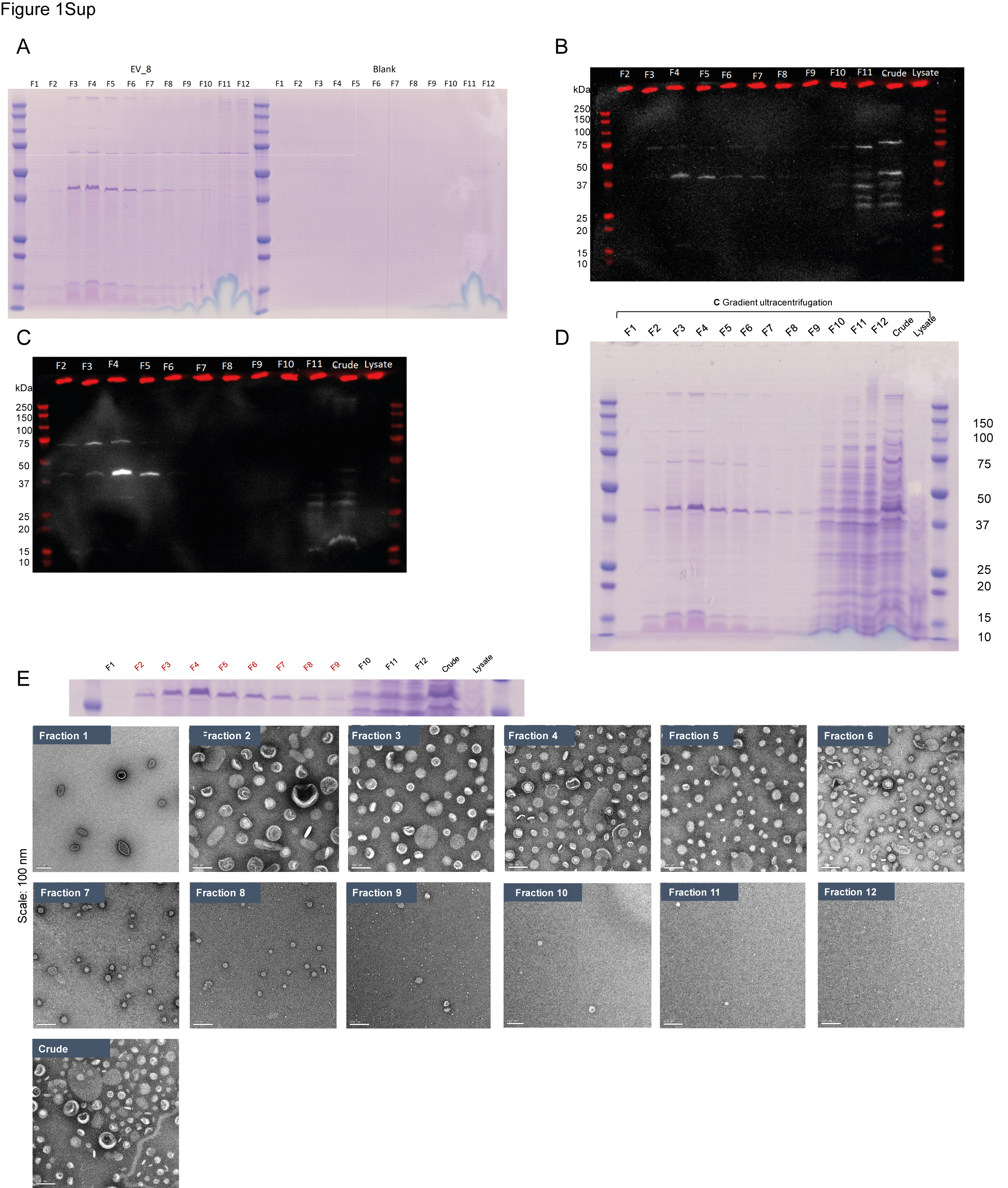

Supplement: Supplementary Figure 1 — F. nucleatum EVs purification and TEM. (A) density gradient fractions total proteins profile of EVs and corresponding blank, stained with Coomassie Blue. (B) Second EVs fractions identified by far-western blot using a FomA specific biotinylated peptide using classical revelation method. (C) Second EVs fractions identified by far-western blot using a FomA specific biotinylated peptide using Femto sensitive revelation method. (D) density gradient fractions total proteins profile of EVs batch corresponding to far-western blot presented in 1SupB and C, stained with Coomassie Blue (E) EVs fractions 1 to 12 imaged by TEM and crude extract (0.1×); size bar, 100nm. [file Image_1.tif]

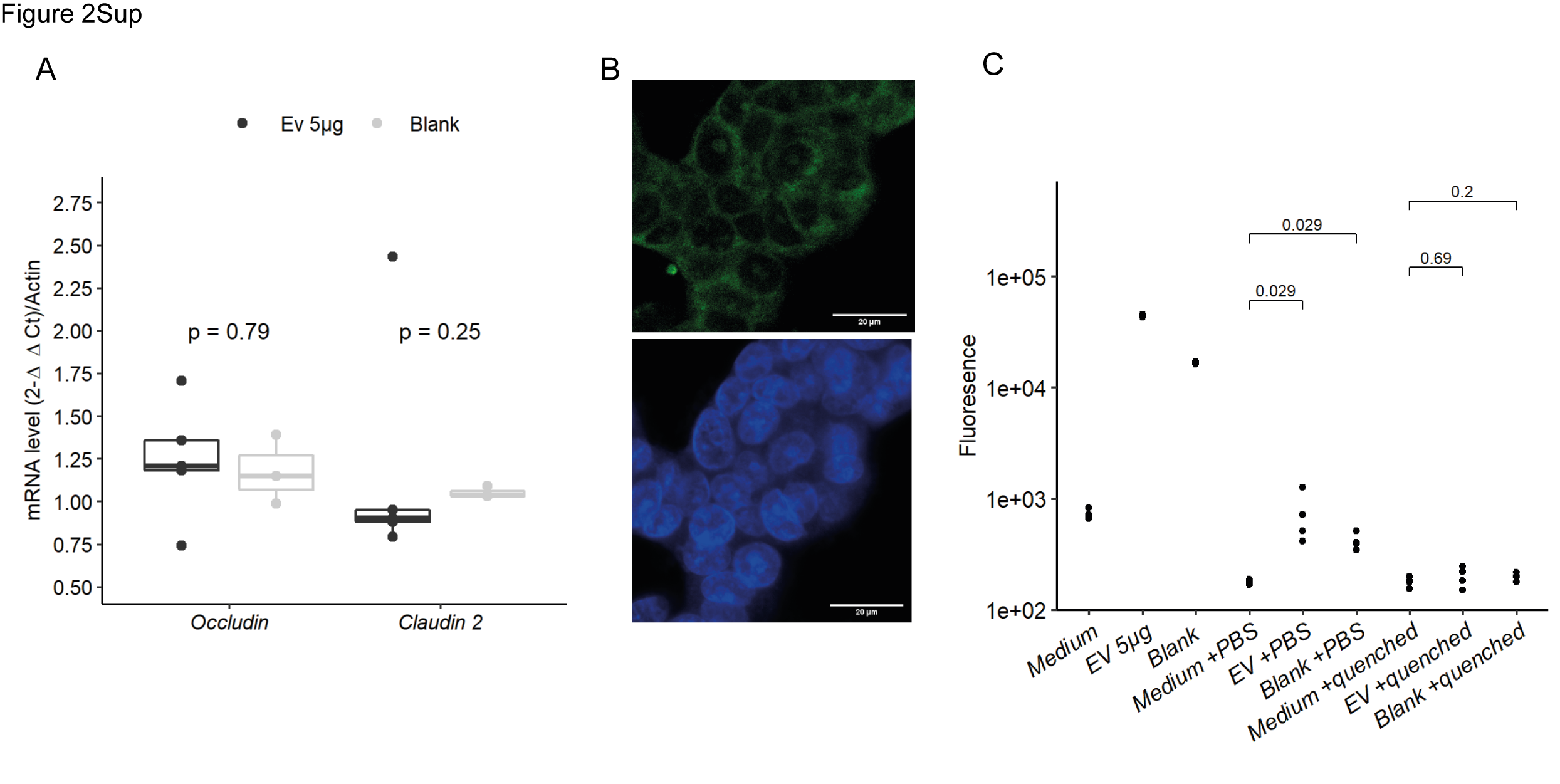

Supplement: Supplementary Figure 2 — (A) Caco-2 differentiated cells were incubated with 5µg of EVs or Blank for 6h before assessing Occludin and Claudin-2 mRNA levels, normalized on Actin (N = 3). (B) FITC-labeled EVs were incubated on HT29 for 6 h, 40X, FITC (Green), nucleus DAPI (Blue). (C) Incubation of 5 µg FITC labeled EVs or Blank for 4 h on T84. Fluorescence level was read after incubation, after PBS washes and after quenching external fluorescence with 0.2% Trypan blue (N = 4). [file Image_2.tif]

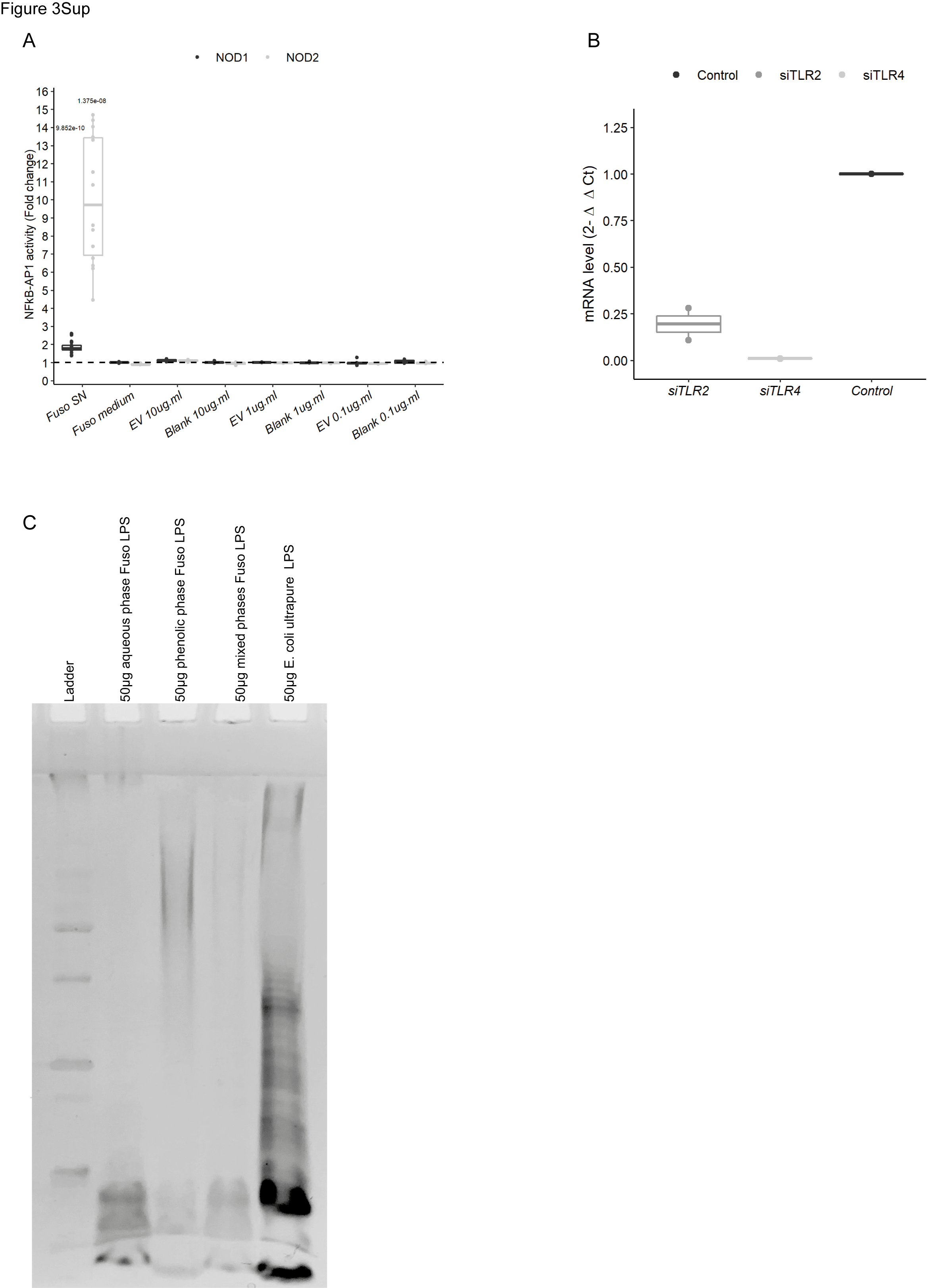

Supplement: Supplementary Figure 3 — (A) Activation of NF-κB-AP1 following EVs exposure of 24 h on HEK-293 NOD1 and NOD2 reporter cell-lines (N ≥ 3) (B) Efficiency of siRNA targeting TLR2 and TLR4 respectively assessed by RT-PCR normalized to GAPDH (N = 2). (C) F. nucleatum LPS extraction on a 12.5% SDS-PAGE gel visualized with Silver stain. Line 1: ladder, Line 2: 50 µg aqueous phase LPS, Line 3: 50 µg phenolic phase LPS, Line 4: 50 µg of mixed (1:1) aqueous and phenolic phase LPS, Line 5: Control (50 µg of E. coli Ultrapure LPS). [file Image_3.tif]
